# Electrolyzed saline coolant mitigates bacterial airborne deposition in dental procedures

**DOI:** 10.3389/froh.2026.1961598

**Published:** 2026-09-25

**Authors:** Haris Munjaković, Tina Mikuletič, Martin Merhar, Naiera Zayed, Marko Kolenc, Damjan Manevski, Tina Triglav, Aleš Fidler, Wim Teughels, Katja Seme, Rok Gašperšič

**Affiliations:** 1 Department of Oral Medicine and Periodontology, University Medical Centre Ljubljana, Ljubljana, Slovenia; 2 Department of Oral Medicine and Periodontology, Faculty of Medicine, University of Ljubljana, Ljubljana, Slovenia; 3 Institute of Microbiology and Immunology, Faculty of Medicine, University of Ljubljana, Ljubljana, Slovenia; 4 Department of Oral Health Sciences, University of Leuven (KU Leuven), Leuven, Belgium; 5 Department of Microbiology and Immunology, Faculty of Pharmacy, Menoufia University, Menoufia, Egypt; 6 Institute for Biostatistics and Medical Informatics, Faculty of Medicine, University of Ljubljana, Ljubljana, Slovenia; 7 Department of Endodontic and Restorative Dentistry, Faculty of Medicine, University of Ljubljana, Ljubljana, Slovenia; 8 Department of Endodontic and Restorative Dentistry, University Medical Centre Ljubljana, Ljubljana, Slovenia; 9 Dentistry Service, University Hospitals Leuven, Leuven, Belgium; 10 Department of Periodontology, Faculty of Dentistry, Chulalongkorn University, Bangkok, Thailand; 11 Department of Restorative Dentistry, Faculty of Dentistry, University Malaya, Kuala Lumpur, Malaysia; 12 University Clinic of Respiratory and Allergic Diseases Golnik, Golnik, Slovenia

**Keywords:** chlorhexidine digluconate, dental aerosol, HOCl, infection control, ultrasonic scaling

## Abstract

**Background:**

Dental procedures generate dental spray that can transmit microorganisms. Substituting conventional coolants with antiseptics may mitigate this risk by preventing the transmission of infectious particles at the source. The aim of this *in vitro* study was to evaluate the efficacy of electrolyzed saline (EOS), a hypochlorous acid (HOCl) based antiseptic as a coolant for mitigating bacterial spray deposition during ultrasonic scaling (USS) in stimulated dental procedure.

**Methods:**

The bactericidal activity of EOS (pH 7.0, 200 ppm) and 0.2% chlorhexidine digluconate (CHX) was tested against *Streptococcus mitis, Streptococcus pneumoniae, Streptococcus pyogenes,* and *Haemophilus influenzae* using a static antibacterial assay. Changes in bacterial morphology were visualized using transmission electron microscopy. A bacteria-laden dental spray model was used to simulate a patient undergoing plaque debridement procedure with ultrasonic scaler in standardized airborne transmission tunnel. Due to safety concerns, pathogen-free consortium of oral streptococci was used. Mitigation of bacterial deposition was quantified using a culture-based method. In addition, the biocompatibility of antiseptics was tested with exposure to human gingival fibroblasts (hGF) and human gingival keratinocytes (hOK).

**Results:**

In the static assay, CHX reduced all tested bacteria species to ≤10 CFU/mL within 10 s of exposure, irrespective of the presence of saliva. In contrast, EOS was generally less effective after 10 s but exhibited near-complete antibacterial activity after 30–60 s, with delayed efficacy in the presence of saliva. During USS procedures, no culturable bacteria were detected in splatter generated with either CHX or EOS. However, compared with saline EOS reduced bacteria-laden droplets/aerosol (CFU/cm^2^) deposition by 92% vs. 81% with CHX (both, *p* < 0.001), with no significant difference between the antiseptics (*p* = 0.607). Both antiseptics effectively reduced bacterial deposition at short range. Nevertheless, only EOS maintained consistent mitigation efficacy over greater distances dominated by aerosol. EOS exhibited minimal cytotoxicity to hGF and moderate effects on hOK, while CHX substantially reduced viability of both cell types.

**Conclusions:**

EOS demonstrated rapid contact-bactericidal activity and comparable dental spray deposition reduction to CHX. As a reasonably biocompatible, broad-spectrum agent, EOS may represent a promising coolant candidate for infection prevention control, warranting further clinical evaluation.

## Introduction

The dental office environment poses a high risk for transmission of bacteria and viruses, a concern that became particularly evident during the COVID-19 pandemic. In addition to respiratory and contact routes, microorganism transmission may also occur via dental spray generated during dental procedures (1).

Bacteria and viruses are present in oral fluids (2, 3) and various respiratory excretions (4) in both symptomatic and asymptomatic patients. Transmission of infectious particles is therefore possible during dental procedures, particularly when using high-speed instruments that can generate dental spray which combines coolant with oral fluids (5), which poses increased risk for oral health providers due to continual exposure to aerosolized bacteria and viruses (6), including antibiotic-resistant strains (7) and important respiratory pathogens (8). The resulting aerosols can persist in the air for extended periods, posing an ongoing infection hazard (9). Therefore, there is a critical need for strategies that can rapidly inactivate microorganisms during dental procedures.

During the COVID-19 pandemic, passive infection control measures such as personal protective equipment, surface decontamination, and air filtration were extensively implemented (10). While essential, these measures cannot fully eliminate the risk of infection transmission. Accordingly, active aerosol mitigation strategies, particularly high-volume evacuation (HVE) (11) and pre-procedural mouth rinses (12), have gained consensus support. HVE effectively removes aerosols before they become airborne but is less efficient against splatter and large droplets (13). Similarly, the antimicrobial activity of pre-procedural rinses, although demonstrated *in vitro* has shown limited translation to clinical efficacy. This inconsistency has been more clearly reported for antiviral activity (14), whereas bacterial mitigation has shown somewhat greater efficacy (15).

Ultimately, it is important to recognize that achieving sterility of the oral cavity is not feasible. Therefore, infection control through disinfecting dental spray has been introduced by substituting standard coolants with antimicrobials. Importantly, aerosol-control approaches are not equivalent to standardized disinfectant efficacy testing, although related, they address distinct research questions. Dental spray mitigation testing requires standardized, procedure-specific protocols, and our established method (16, 17) provides standardized *in vitro* framework for assessing microbial mitigation in dental spray.

Recently, Electrolyzed saline (EOS), a chlorine-based antimicrobial with hypochlorous acid (HOCl) as its main antimicrobial component, has gained increased attention as a safe, fast-acting disinfectant suitable for use in oral environment. *in vitro* evidence indicates that EOS can effectively mitigate the dental spray transmission of both enveloped and non-enveloped viruses (17, 18). Alongside the widely studied chlorhexidine digluconate (CHX) (19), EOS may, through its potent oxidizing activity and broad-spectrum antibacterial properties (20), help overcome the limitations of current infection control measures.

Building on this rationale, we conducted an *in vitro* study to evaluate the efficacy of EOS as a coolant for mitigating bacterial spray deposition generated by an ultrasonic scaler during a simulated dental procedure. We hypothesized that replacing standard saline coolant with EOS during ultrasonic scaling (USS) would reduce bacterial deposition in dental spray. The null hypothesis was that EOS would not differ from standard saline coolant in recovered bacterial load (CFU/cm^2^), whereas the alternative hypothesis was that EOS would reduce the recovered bacterial load.

## Materials and methods

A detailed description of the growth conditions of all cell lines and bacterial strains growth conditions is provided in the Supplementary Appendix.

### Study design and model bacteria used

The study was conducted in sequential experimental stages to evaluate both the antibacterial efficacy of the tested antiseptics and their ability to mitigate dental spray mediated bacterial deposition using a controlled *in vitro* USS simulation model.

CHX (0.2%, w/v) and EOS (pH 7.0, 200 ppm) were evaluated for their antibacterial activity, with saline (NaCl 0.9%) serving as the negative control.

First, static suspension assays were performed to determine the bactericidal efficacy of the antiseptics against *Streptococcus mitis*, a commensal oral bacteria, and respiratory pathogens that may be present in saliva, including *Streptococcus pneumoniae*, *Streptococcus pyogenes*, and *Haemophilus influenzae*. Transmission electron microscopy (TEM) was additionally used to assess antiseptic-induced ultrastructural changes in bacterial cells. These organisms were included because they may represent potential sources of infectious contamination in dental spray.

Second, dental spray mediated bacterial deposition was evaluated using an *in vitro* experimental model simulating supragingival plaque debridement with an ultrasonic scaler. For safety reasons, the respiratory pathogens used in the static assays were not introduced into the dental spray model. Instead, a pathogen-free multispecies culture comprising *Streptococcus mitis*, *Streptococcus oralis*, *Streptococcus gordonii*, *Streptococcus salivarius*, *Streptococcus sobrinus*, and *Streptococcus mutans* was selected to mimic the oral microbiota (21). The potential for antiseptic-mediated mitigation of bacterial deposition was assessed by substituting the ultrasonic scaler coolant with one of the tested antiseptics and comparing bacterial dissemination with that observed under the control condition.

Additionally, the biocompatibility of the antiseptics was tested using human oral keratinocytes (hOK) and human gingival fibroblasts (hGF).

### Preparation of antiseptics, artificial saliva and neutralization solution

Sterile saline (NaCl 0.9%, w/v) was obtained from (B. Braun). CHX (20%, w/v) was obtained from (Sigma-Aldrich) and diluted with Milli-Q® water to a final concentration of 0.2% (w/v). A sterile saline (B. Braun) was used to prepare EOS using a EOS generating device (Zarsten) according to the manufacturer's instructions. The free chlorine concentration was checked using spectrophotometric analysis (Hanna Instruments). Free chlorine concentrations were adjusted to 200 ppm by dilution with Milli-Q® water, and the pH was adjusted to 7.0 (corresponding to an HOCl:OCl^−^ ratio of 75:25) using HCl. Independent batches were prepared before each experiment, filter sterilized and stored at room temperature for the duration of the experiment.

Artificial saliva was prepared as described per Wasik et al. (22) by dissolving Na_2_HPO_4_ (0.6 g/L) (Merck), anhydrous CaCl_2_ (0.6 g/L) (Merck), KCl (0.4 g/L) (Sigma-Aldrich), NaCl (0.4 g/L) (Sigma-Aldrich), Urea (4.0 g/L) (Merck), Mucin (2.0 g/L) (Roth) in Milli-Q® water. The pH was adjusted to 7.2 using NaOH. Artificial saliva was sterilized and stored at 4 °C.

Neutralizing solution was prepared as described per (23) by dissolving Saponin (30 g/L) (Sigma-Aldrich), Lecithin (3 g/L) (Sigma-Aldrich), Tween 80 (30 g/L) (Merck), Na _2_S _2_O _3_· (H _2_O)_5_ (7.8 g/L) (Merck), L-histidine (1 g/L) (Sigma-Aldrich) in Milli-Q® water. Neutralizing solution was filter sterilized and stored at 4 °C.

### Antibacterial assay

Antimicrobial efficacy of the antiseptics was evaluated by a quantitative suspension method. Overnight bacterial suspensions of *S. mitis*, *S. pneumoniae*, *S. pyogenes*, and *H. influenzae* were adjusted to 0.5 McFarland standard, and 1 mL was centrifuged for 5 min at 5,000× g. The pelleted cells were resuspended in 100 µL of saline and mixed with 900 µL of antiseptic (final concentration: CHX 0.2%, EOS 200 ppm or saline control) for 10, 30, or 60 s. After the designated exposure time, 500 µL of neutralization solution was added to stop the reaction and rule out post-sampling carry-over disinfection. Bacterial load reduction was assessed by culturing the bacteria on appropriate agar media for each (blood agar or chocolate blood agar plates) to determine CFUs after 48 h of incubation in 5% CO_2_ at 37 °C. In addition, the effect of interference between organic matter and antiseptic was tested by preparing the bacterial suspension with 100 µL of artificial saliva before exposure to the antiseptic. The assays were performed in three technical and three biological replicates.

### Transmission electron microscopy

The treated bacterial samples were fixed immediately after exposure to antiseptic, directly on formvar-coated grids. Negative staining was performed with 0.5% or 1% phosphotungstic acid. The grids were examined using a TEM (JEM-1400 Plus; JEOL) at 120 kV. Bacterial cell morphology was assessed qualitatively. The extent of damage to the bacterial morphology was assessed by comparing the effects of the specific antiseptic with the phosphate-buffered saline (PBS) negative control. The samples were prepared from three biological replicates, with at least five images acquired per replicate. Representative images illustrating the observed effect were selected by a trained microscopist. The assessment was not blinded.

### Bacteria-laden dental spray deposition set-up

In this study, the experimental set-up developed by Fidler et al. (2022) to test microbial spread by USS in transmission tunnel was slightly modified. Specifically, the additional cyclone air collection was excluded. Briefly, a transmission tunnel (Acrytech) was used to ensure standardize conditions and steady airflow for all replicates. A piezoelectric ultrasonic scaler (Varios 970, NSK-Dental-Nakanishi) with a handpiece (VA2-LUX-HP, NSK-Dental-Nakanishi) and a general prophylaxis tip (G5, NSK-Dental-Nakanishi) was used to generate the dental spray. The ultrasonic scaler was positioned on a platform, 25 cm above the transmission tunnel floor (Figure 1). The coolant flow rate was set to 17.5 mL/min. The ultrasonic scaler tip was positioned in a semi-cylindrical groove, allowing for free oscillation and uniform generation of dental spray. The multispecies bacterial suspension was delivered separately via an infusion administration set into the same groove at a flow rate of 1.73 mL/min to facilitate the generation of a bacteria-laden dental spray.

**Figure 1:**
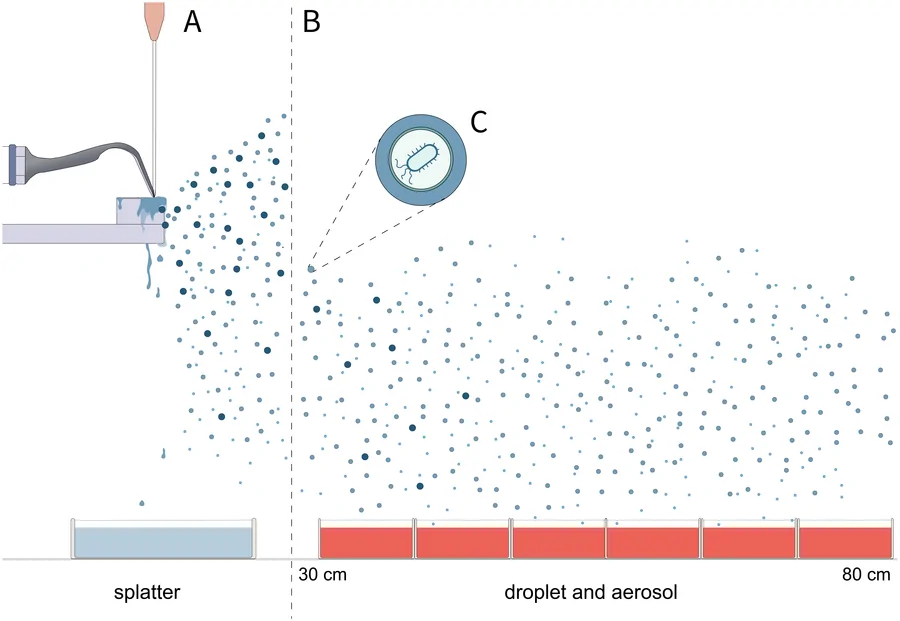
A bacteria-laden dental spray deposition model simulating an ultrasonic scaling (USS) procedure was used. Mitigation was tested by replacing the USS coolant with antiseptics. Sampling included: **(A)** splatter (coolant mixed with bacterial suspension) collected under the spray source; **(B)** droplets and aerosol collected on blood agar plates placed 30–80 cm (contamination surface) from the spray source; and **(C)** bacteria-laden droplet/aerosol.

Multispecies bacterial suspension was prepared by combining equal volumes of overnight cultures (McFarland standard = 4.5) of *S. mitis*, *S. oralis*, *S. gordonii*, *S. salivarius*, *S. sobrinus*, and *S. mutans*. 1 mL of each culture was added to 50 mL of sterile saline resulting in a bacterial suspension with a final concentration of 8 log_10_ CFU/mL, corresponding to the upper range of bacterial concentrations typically found in saliva (24). Bacterial deposition was achieved by simultaneous activation of the ultrasonic scaler and flow of the bacterial suspension over a period of 5 min. To evaluate the antibacterial effect the ultrasonic scaler cooling liquid was replaced with one of the antiseptics.

### Evaluation of bacterial deposition prevention

Bacterial sampling during the dental spray deposition test was divided into two sections: splatter and droplet/aerosol samples (Figure 1) (1). The splatter sample consisted of a mixture of coolant fluid and bacterial suspension that dripped freely from the groove and was collected directly under the generating platform_._ After the procedure, splatter samples were serially diluted in PBS and plated for CFUs quantification_._ The plates with splatter samples were incubated for 48 h in 5% CO_2_ at 37 °C. The assays were performed in one technical and three biological replicates. (2) The droplet/aerosol sample was collected on blood agar plates placed 30 to 80 cm from the ultrasonic scaler tip during the 5 min active spraying procedure, followed by a 5 min settling period. The droplet/aerosol samples (blood agar plates) were incubated for 48 h in 5% CO_2_ at 37 °C. After incubation, plates were photographed (prior orientation marked) to record the location of bacterial colonies. The total surface contamination area was divided into 30 × 24 (rows × columns) equal fields to increase spatial resolution in the analysis. Colony location and density were quantified using ImageJ (NIH) to determine CFU/cm^2^. The assays were performed in one technical and three biological replicates.

### Biocompatibility assay

The antiseptic solutions were tested for biocompatibility using the human oral keratinocyte cell line (hOK-18A) cultured as described by (21) and the human gingival fibroblasts cell line (hGF-1). For the cytotoxicity assay, confluent hOK-18A and hGF-1 cells were exposed to the tested antiseptic concentrations and their serial (1:2) dilutions in serum-free medium for 1 min (17). After removal of the test solutions, an XTT-assay (Roche) was performed according to the manufacturer's instructions. The assays were performed in three technical and three biological replicates.

### Statistics

Antibacterial and biocompatibility assay results were analysed using two-way analysis of variance (ANOVA), followed by Tukey's *post hoc* test for multiple comparisons.

For the dental spray bacterial deposition study, the primary outcome, bacterial deposition on agar plates (CFU/cm^2^) was analysed using multiple linear regression. The model included antiseptic type, distance from the source of dental spray, and sampling position (centre, right, left) as independent variables. Effect size and percentage reduction were derived from a simpler, more stable main-effects model. An additional interaction model was fitted to assess whether the effect of antiseptic type varied with distance from the dental spray source. Regression coefficients are reported with 95% confidence intervals. Statistical significance was defined as *p* < 0.05. Analyses were performed using GraphPad Prism 9.

As a secondary outcome, the number of the contaminated fields was analysed using a generalized linear mixed model with logit link function. The outcome is thus a binary variable (whether a field is contaminated or not), whereas antiseptic type and distance from the source of the dental spray were included as fixed-effect covariates, with replication as a random effect (random intercept). An interaction term between antiseptic type and distance was also included to provide better interpretation. Due to quasi-complete separation of the outcome (i.e., for some antiseptics, few/many wells were contaminated) we used a penalized estimation approach (25). Model effects were expressed as odds ratios (ORs) with 95% confidence intervals. The analysis was conducted using R (v4.4.2).

## Results

### Antibacterial assay

CHX demonstrated rapid and robust bactericidal activity, reducing all tested bacterial species bellow the limit of detection (LOD) (≤10 CFU/mL) within 10 s of exposure, irrespective of the presence or absence of artificial saliva as an organic burden (*p* < 0.001). In the absence of artificial saliva, EOS also demonstrated rapid bactericidal effect, reducing *S. mitis* and *H*. *influenzae* to ≤LOD and achieving ≥99.98% reduction of other tested bacteria within 10 s (*p* < 0.001). Reduction of *S. pneumoniae* to ≤LOD required 30 s of exposure, whereas *S. pyogenes* reached 25 (±4) CFU/mL after 60 s of contact time. In the presence of artificial saliva, the bactericidal activity of EOS was delayed. Despite this, ≥99.92% reductions were observed within 10 s for all species (*p* < 0.001), although effect remained significantly lower compared to CHX. By 60 s, bacterial counts ranged from ≤LOD to 12.3 (±34) CFU/mL (*p* < 0.001), indicating high antibacterial activity of EOS (Figure 2 and Supplementary Table 1).

**Figure 2:**
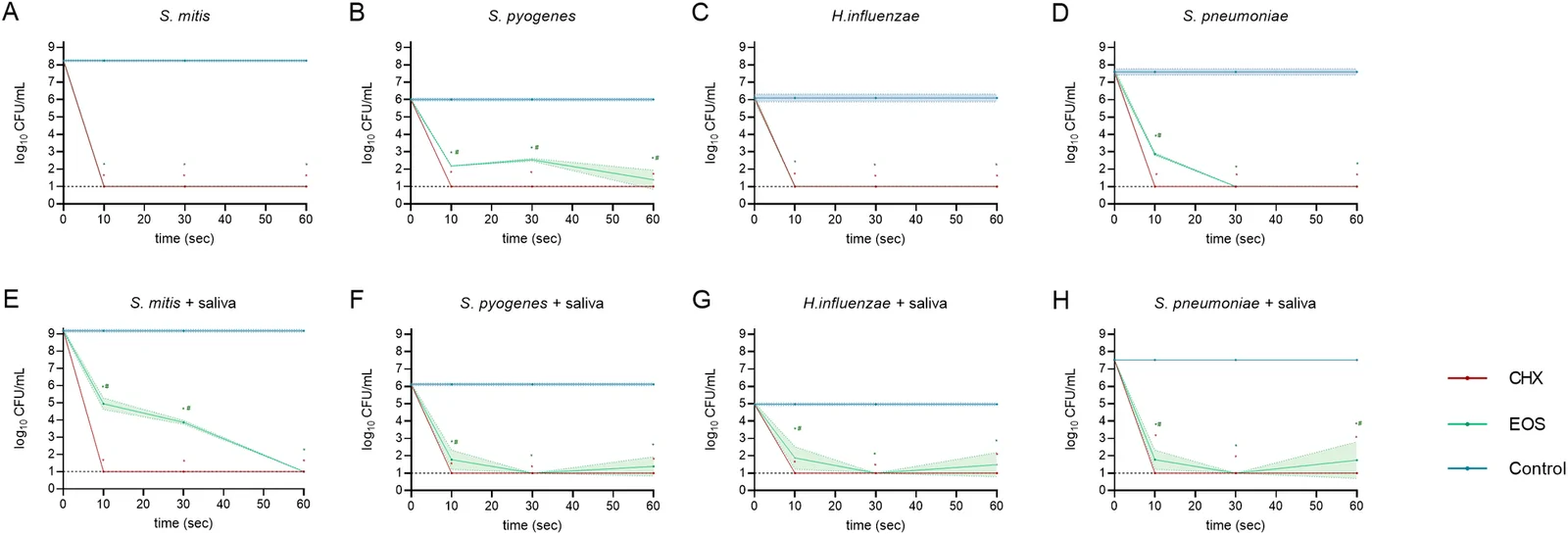
Efficacy and of the antiseptics in reducing the bacterial loads of *Streptococcus mitis*, *Streptococcus pneumoniae*, *Streptococcus pyogenes*, and *Haemophilus influenzae* treated with saline (control), chlorhexidine digluconate (CHX), and electrolyzed saline (EOS) for 10, 30, and 60 s in the absence **(A–D)** or presence of artificial saliva **(E–H)**. Values are presented as mean ± SD. Dashed line indicates limit of detection (LOD) (≤10 CFU/mL). *Indicates significant difference from saline (*p* ≤ 0.05); ^#^– indicates significant difference from CHX (*p* ≤ 0.05).

### Transmission electron microscopy

Transmission electron microscopy was used to qualitatively assess morphological changes in the tested bacterial species following treatment with EOS, and CHX. PBS treatment was used as a control (Figure 3). Bacteria in the PBS group retained their characteristic morphology, with intact, continuous cell walls and no evidence of membrane discontinuity or leakage. In contrast, exposure to EOS resulted in consistent ultrastructural alterations. All bacterial species showed varying degrees of cell envelope disruption, and extracellular granular material, including DNA, was observed, suggesting leakage of cytoplasmic components. CHX treated cells exhibited membrane detachment, gaps between the cytoplasmic membrane and cell wall, and cellular shrinkage, thus loss of cell shape. The extent of both EOS and CHX-induced damage was comparable between Gram-positive (*S. mitis*, *S. pneumoniae*, and *S. pyogenes)* and Gram-negative (*H. influenzae*) species, indicating a uniform broad-spectrum disruptive effect.

**Figure 3:**
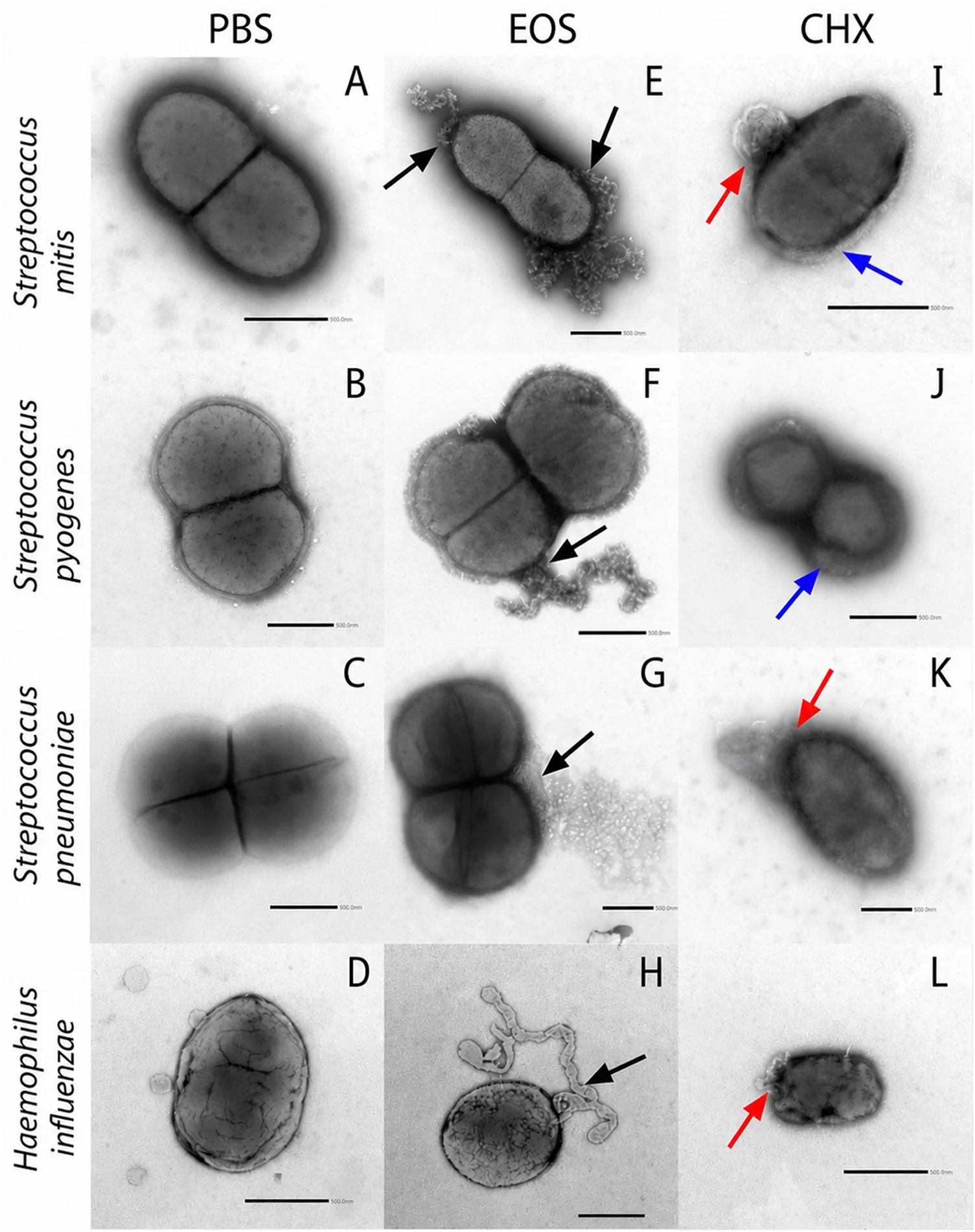
Transmission electron microscopy (TEM) of bacterial ultrastructural alterations following treatment with electrolyzed saline (EOS) or chlorhexidine digluconate (CHX). Representative TEM micrographs of *Streptococcus mitis*, *Streptococcus pyogenes*, *Streptococcus pneumoniae*, and *Haemophilus influenzae* after exposure to phosphate-buffered saline (PBS; Control), EOS, or CHX. PBS-treated bacterial cells exhibit normal morphology with intact, well-defined cell walls and uniform cytoplasmic density [Panels **(A–D)**]. EOS treatment causes pronounced cell-envelope disruption and partial leakage of intracellular material, including DNA in all species [black arrows; Panels **(E–H)**]. CHX exposure resulted in severe morphological alterations, including loss of cellular shape and cell wall integrity [red arrows; Panels **(I, K, and L)**], and distinct gaps between the cytoplasmic membrane and cell wall [blue arrows; Panels **(I and J)**]. Cellular shrinkage was also evident. Scale bars: 500 nm.

### Droplet and aerosol spread

Concentration of bacteria in the test suspension was 7.6 (±0.1) log_10_ CFU/mL. Splatter collected during the control procedure contained 6.4 (±0.2) log_10_ CFU/mL, corresponding to an approx. 1:10 dilution effect of the USS procedure. In contrast, no culturable bacteria were detected under the experimental conditions from splatter generated with either CHX or EOS, indicating high antimicrobial activity.

Model estimates for bacterial colonies detection (non-contaminated/contaminated area) at the spray source (distance = 0; 30 cm) shown that, the odds of detecting bacterial contamination were significantly lower for CHX and EOS compared with the control (Figures 4A–C, Supplementary Table 2). In contrast to the chlorine-containing antiseptic EOS, CHX was not as effective in inactivating the bacteria in dental spray. Nevertheless, the estimated odds ratios (ORs) for contamination when using antiseptic coolants were close to zero, due to quasi-complete separation among antiseptic types. The OR for the intercept was large, as all fields were contaminated when saline was used. Distance did not have a statistically significant effect on the odds of contamination for saline. To better assess the effect of distance for CHX and EOS, we computed their combined slopes and found out that distance is associated with an increasing OR of contamination in the CHX group [OR = 1.42; 95% CI (1.38, 1.46)], and a decreasing OR in the EOS group [OR = 0.75; 95% CI (0.66, 0.85)]. The estimated standard deviation of the random effect was small [OR = 0.09; 95% CI (0.01, 0.67)], indicating low variability across replications.

**Figure 4:**
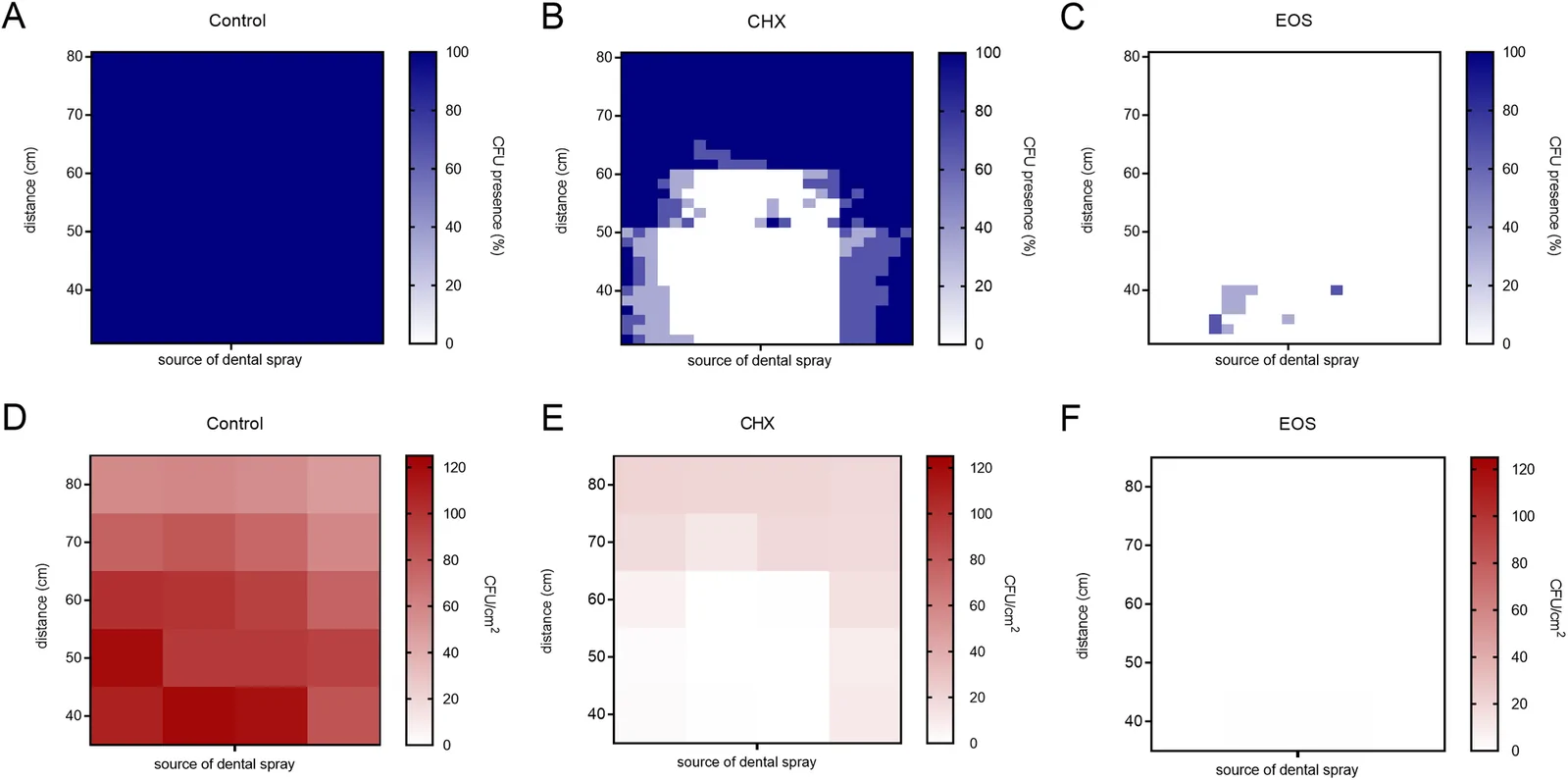
Spatial representation of dental spray bacterial deposition assay during a simulated dental procedure using ultrasonic scaler. Panels **(A–C)** show the presence of non-contaminated and contaminated areas for saline (Control), chlorhexidine digluconate (CHX), and electrolyzed saline (EOS) coolant, represented on a blue gradient scale, expressed as percentages. Panels **(D–F)** show quantification of bacterial load (CFUs/cm^2^) for saline (Control), CHX, and EOS coolant. Values are presented on a red gradient scale, expressed as means. SD are not shown for clarity and readability of the figure. SD for quantification of bacterial load is provided in Supplementary Figure 1.

Multiple linear regression showed that antiseptic type and distance from the dental spray source were significant factors influencing bacterial load, while sampling position had no effect. Baseline bacterial presence (Control) was 93.6 CFU/cm^2^, 95% CI [85.5, 101.6], with colonies observed across the entire surface and gradually decreasing with distance. CHX reduced bacterial load by −76.2 CFU/cm^2^, 95% CI [−83.4, −68.9] (≈81% reduction), while EOS reduced it by −85.7 CFU/cm^2^, 95% CI [−92.9, −78.5] (≈92% reduction), demonstrating that both antiseptics were highly effective (*p* < 0.001), with EOS achieving a greater reduction, although the difference between CHX and EOS was not statistically significant (*p* = 0.607). Increasing distance further decreased bacterial counts, whereas central, right, and left sampling positions showed no significant differences (Figures 4D–F, Supplementary Table 3).

To further assess whether antiseptic efficacy varied with distance, an interaction model was fitted (Supplementary Table 4). In the control condition, CFU/cm^2^ decreased by 3.40 per unit distance (*p* < 0.001). In the presence of CHX, this distance-related decline resulted in a net increase of approximately +1.22 CFU/cm^2^ per unit distance (*p* < 0.001), consistent with re-emergence of detectable CFU at greater distances. In contrast, EOS showed a neutral distance effect +0.03 CFU/cm^2^ per unit distance (*p* < 0.001) indicating stable suppression. Together, these findings suggest that while both antiseptics markedly reduce bacterial deposition at short range, where the distribution of dental spray results in greater contact time (e.g., more droplets), EOS maintains more consistent mitigation across increasing distances, where aerosolized particles predominate, whereas CHX exhibits a modest distance-dependent attenuation. Nevertheless, both antiseptics remain highly effective in mitigating bacterial spread.

### Biocompatibility assay

The cytotoxicity of the tested antiseptics and their serial dilutions (up to 1:32) was evaluated using hOK-18A and hGF-1 monolayers (Figure 5, Supplementary Table 5). Exposure of hGF cells to EOS did not affect cell viability compared with the negative control. In hOK cells, EOS exhibited a moderate cytotoxic effect at concentrations up to a 1:4 dilution, resulting in up to ≈35% reduction in cell viability. In contrast, CHX significantly reduced the viability of both hGF and hOK at nearly all dilutions, except for the 1:32 dilution for hGF.

**Figure 5:**
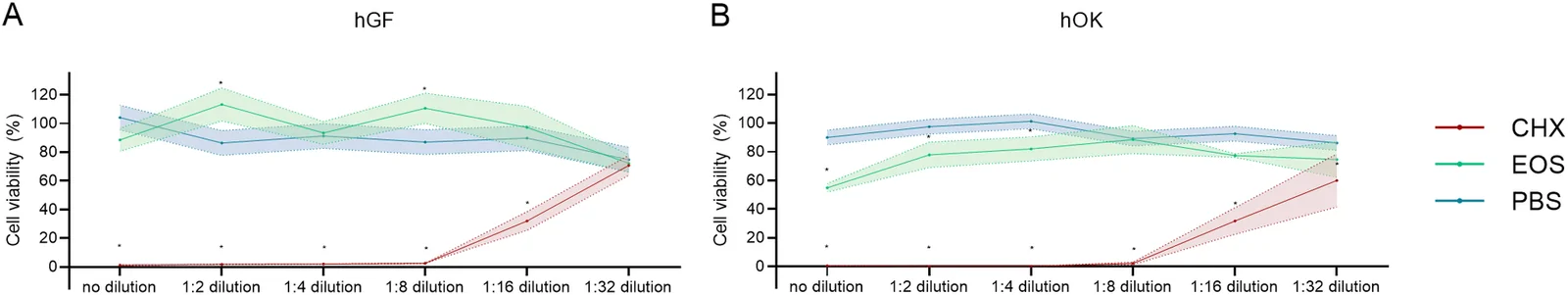
Biocompatibility of **(A)** human gingival fibroblasts (hGF); and **(B)** human oral keratinocytes (hOK) monolayers after exposure to different dilutions of antiseptics: phosphate buffered saline (PBS), chlorhexidine digluconate (CHX), and electrolyzed saline (EOS) for 1 min, expressed as percentages. Values are presented as mean ± SD. *indicates significant difference from PBS, (*p* ≤ 0.05).

## Discussion

This *in vitro* study examined the use of antiseptics (CHX and EOS) as coolants during simulated USS procedures to substantially reduce airborne bacterial deposition in a standardized experimental setup. Given the increasing prevalence of immunosuppressed individuals (26), the risk of infection in contexts such as the COVID-19 pandemic (27), the presence of opportunistic human pathogens in dental unit waterlines (28), and emerging infectious diseases, the outcomes of this study may have implications for dental infection control. Notably, both antiseptics, EOS and CHX substantially reduced culturable bacterial deposition generated during USS. Although EOS showed numerically greater and distance independent reduction profile relative to CHX, the direct comparison was not statistically significant.

CHX has a long-standing tradition as the most versatile broad-spectrum antimicrobial agent in periodontal therapy. However, it has serious drawbacks, including the potential to induce dysbiosis (29), antibiotic cross-resistance (30), and impairment of the oral microbiota nitrate reducing capacity (31). In contrast, EOS has demonstrated beneficial modulatory effects on *in vitro* subgingival biofilm ecology, supporting biofilm resilience, by preventing dysbiosis (32) and has been proven safe antimicrobial in a recent RCT where EOS was tested as a mouthrinse in healthy population (33). Complementary evidence from *ex vivo* biofilm formation (34) and *in vivo* rat periodontitis model (35) further support HOCl as beneficial agent in selective biofilm recolonization with preservation of microbiota balance resulting in lower inflammation and inhibition of bone resorption.

It is important to note that the beneficial effect of HOCl on the biofilm is primarily driven by biofilm dynamics and differs from its role in preventing airborne microbial transmission. Biofilms are more complex and robust than individual cells or aggregates found in dental spray. HOCl acts as a non-specific oxidizing agent that perturbs natural biofilm development, which normally progresses toward anaerobiosis and a complex metabolic network. This disruption of biofilm niche, driven by high redox potential resulting in oxidative stress, with a more pronounced effect on anaerobic Gram-negative bacteria may promote beneficial recolonization favouring commensal bacteria (33). In contrast, its effect on individual microbial particles primarily results in direct contact-mediated inactivation.

Consequently, both CHX and EOS have been used in this study as candidate antiseptic coolants for evaluation. To better reflect a clinically relevant scenario, the contact time between bacteria and the coolant in our airborne transmission model was limited to less than one second during dental spray formation and settling (36), except in the immediate vicinity of the spray source, where higher droplet accumulation, attributable to ballistic principles, likely resulted in prolonged exposure.

In this study, CHX was not susceptible to inactivation by organic matter, and no culturable bacteria were detected under the experimental conditions after 10 s of contact in static assay. However, it only partially reduced bacterial contamination under sub-second exposure in deposition assay, which is relevant for evaluating airborne prevention. This discrepancy highlights the importance of using appropriate dynamic models when assessing mitigation of aerosol deposition. Despite this limitation, CHX remains clinically relevant due to its ability to bind to bacterial cell walls and membranes, exhibiting a residual effect on oral surfaces. Consistently, *in vivo* studies have shown that CHX reduces bacterial dental spray viability by 61%–70% when used as pre-procedural rinse (15, 37), and 59%–78% when used in coolant form (19, 38), a level of mitigation comparable to the 81% reduction demonstrated with our model. This parallel outcome further validates the adequacy of our *in vitro* deposition assay model, supporting its translational relevance and reinforcing the rationale for considering EOS alongside CHX.

Regarding EOS, it proved effective, almost completely preventing bacterial spread, with a 92% reduction. Moreover, its efficacy was not distance dependent, indicating a consistent contact-mediated antimicrobial deactivation. Its efficacy was comparable to that of CHX, which also produced a significant, though incomplete, reduction in bacterial load. EOS showed additional advantages, exhibited higher biocompatibility compared to CHX, with hOK cells being more susceptible than hGF. More importantly, our recent *in vitro* evidence indicates that EOS in a form of a coolant can effectively mitigate the dental spray transmission of both enveloped and non-enveloped viruses (17).

As with CHX, EOS deposition assay results differ from static antibacterial studies, where EOS required up to 60 s to exert its disinfection action in the presence of saliva, likely due to reduced availability of active free chlorine. Yet, our previous findings suggest that EOS is more effective under dynamic conditions than predicted by static assays (17). The continuous flow of coolant in our model, simulating clinical procedure (approximately 1:10 dilution) (16), likely maintains high antimicrobial activity by replenishing active chlorine components and compensate for HOCl inactivation by organic matter in the oral cavity.

Differences in mechanisms of action may explain the observed performance differences among disinfectants. EOS is a chlorine-based antimicrobial agent whose primary active component, HOCl, acts as a potent oxidizing agent. The broad-spectrum antibacterial effects of EOS result from its ability to target multiple microbial components, including disruption of the bacterial cell envelope (39), denaturation of proteins, and degradation of nucleic acids (40), leading to multifaceted oxidative and contact inactivation of bacteria. This mode of action corresponds with structural damage observed by TEM imaging, which are quintessential for immediate inactivation of microbial particles, a critical attribute when preventing airborne transmission. However, these findings highlight the potential of both EOS and CHX as reasonable adjuncts to current infection control measures, warranting clinical evaluation for mitigating airborne transmission in dental settings.

Considering the growing global burden of antimicrobial resistance (AMR) (41), chlorine-based agents are no exception. Despite their potential beneficial role in preventing dysbiosis, extensive use can induce antibiotic resistance (42, 43). These considerations highlight the need to carefully weigh the risk-benefit profile of chemical antimicrobials and emphasise their complementary role to mechanical mitigation strategies such as HVE, which reduce aerosol spread without contributing to resistance development. However, it remains important to be mindful of the inherent limitations of both approaches.

This study has several limitations. The dental spray deposition experiment relied on an *in vitro* setup using a simplified, pathogen-free bacterial community composed of commensal oral microbiota representatives for safety reasons, which may not accurately reproduce the biochemical, rheological, or clinical conditions associated with fully biohazardous bacterial aerosol spread. The artificial experimental conditions and absence of real human aerosol heterogenicity, including host-derived cells, proteins, and complex particle size distributions, may also influence the inactivation dynamics observed. In addition, the agar-plate collection method does not distinguish between bacteria deposited through larger droplets and aerosol, limiting the ability to separately assess droplet and aerosol deposition. The relatively limited number of independent replicates also warrants caution in generalizing the findings. Furthermore, study-specific validation of the neutralization procedure including confirmation of complete antiseptic quenching and absence of neutralizer toxicity, in static antibacterial assay was not performed. The absence of clinical outcome data limits the translational value of the findings. Moreover, atomization varies among dental instruments which may lead to performance variability across commercially available systems. Biocompatibility was assessed only in short-term *in vitro* cell-line assay and does not establish clinical tolerability or safety. Finally, although prolonged use may carry certain risks (44), neutral pH EOS has been shown to be minimally corrosive to dental appliances (20) and has demonstrated antibiofilm efficacy in dental unit water systems (45).

## Conclusion

Nevertheless, *in vitro* findings suggest that substituting standard dental coolants with EOS, a chlorine-based antiseptic, may help reduce viral (16, 17) and bacterial deposition during simulated USS procedure. As a rapid, broad-spectrum antibacterial agent with a favourable biocompatibility profile, EOS unlike CHX does not carry the same concerns and appears comparable to established disinfectants in dentistry. However, despite its strong potential, clinical trials are required to confirm its efficacy and safety as an adjunct to standard infection control measures.

## Data Availability

The original contributions presented in the study are included in the article/Supplementary Material, further inquiries can be directed to the corresponding author/s.
