# Supplementary material for: Electrolyzed saline coolant mitigates bacterial airborne deposition in dental procedures
Source: Front Oral Health. 2026 Sep 25;7:1961598. doi: 10.3389/froh.2026.1961598 (PMC13651463; doi:10.3389/froh.2026.1961598)
Supplement: Supplementary file 1 [file Supplementaryfile1.docx]

**SUPPLEMENTARY APPENDIX**

Electrolyzed Saline Coolant Mitigates Bacterial Airborne Deposition in Dental Procedures

Haris Munjaković^1,2^, Tina Mikuletič^3^, Martin Merhar^1,2^, Naiera Zayed^4,5^, Marko Kolenc^3^, Damjan Manevski^6^, Tina Triglav^3^, Aleš Fidler^7,8^, Wim Teughels^4,9,10,11^, Katja Seme^12^, and Rok Gašperšič^1,2*^

^1^ Department of Oral Medicine and Periodontology, University Medical Centre Ljubljana, Ljubljana, Slovenia

^2^ Department of Oral Medicine and Periodontology, Faculty of Medicine, University of Ljubljana, Ljubljana, Slovenia

^3^ Institute of Microbiology and Immunology, Faculty of Medicine, University of Ljubljana, Ljubljana, Slovenia

^4^ Department of Oral Health Sciences, University of Leuven (KU Leuven), Leuven, Belgium

^5^ Department of Microbiology and Immunology, Faculty of Pharmacy, Menoufia University,

^6^Institute for Biostatistics and Medical Informatics, Faculty of Medicine, University of Ljubljana, Ljubljana, Slovenia

^7^ Department of Endodontic and Restorative Dentistry, Faculty of Medicine, University of Ljubljana, Ljubljana, Slovenia

^8^ Department of Endodontic and Restorative Dentistry, University Medical Centre Ljubljana, Ljubljana, Slovenia

^9^ University Hospitals Leuven, Dentistry service, Leuven, Belgium

^10^ Chulalongkorn University, Faculty of Dentistry, Department of Periodontology, Bangkok, Thailand

^11^ University Malaya, Department of Restorative Dentistry, Faculty of Dentistry, Kuala Lumpur, Malasia

^12^ University Clinic of Respiratory and Allergic Diseases Golnik, Golnik, Slovenia

* Address correspondence to Rok Gašperšič, [rok.gaspersic@mf.uni-lj.si](mailto:rok.gaspersic@mf.uni-lj.si)

**Materials and methods**

**Cell line growth conditions**

The human gingival fibroblasts (hGF) were cultured in DMEM:Ham’s F12 medium (CLS Cell Lines Services) supplemented with 10% fetal bovine serum (FBS) (EuroClone), 10 ng/mL fibroblast growing factor (Merck), and 10 µg/L Insulin (Merck). The human oral keratinocyte (hOK) cell line was grown using keratinocyte medium (Gibco), supplemented 10 % FBS (EuroClone), bovine pituitary extract (25 µg/mL) (Gibco) and recombinant epidermal growth factor (5 ng/mL) (Gibco). For biocompatibility assay, 96-well plates with a density of 10^5^ cells/mL were prepared. The experiments were conducted on 48-hour cell monolayers.

**Bacteria strains growth conditions**

| **Bacterial strain** | **Incubation conditions** | **Culture maintenance** |
| --- | --- | --- |
| *Streptococcus pyogenes* ATCC 19615 | 5% CO_2_ | blood agar |
| *Streptococcus pneumoniae* ATCC 49619 | 5% CO_2_ | blood agar |
| *Haemophilus influenzae* ATCC 49247 | 5% CO_2_ | chocolate blood agar |
| *Streptococcus sobrinus* ATCC 20742 | 5% CO_2_ | blood agar supplemented with hemin (5.0 µg/mL), menadione (1.0 µg/mL) |
| *Streptococcus oralis* DSM 20627 | 5% CO_2_ | blood agar supplemented with hemin (5.0 µg/mL), menadione (1.0 µg/mL) |
| *Streptococcus mitis* DSM 12643 | 5% CO_2_ | blood agar supplemented with hemin (5.0 µg/mL), menadione (1.0 µg/mL) |
| *Streptococcus sanguinis* LM 14657 | 5% CO_2_ | blood agar supplemented with hemin (5.0 µg/mL), menadione (1.0 µg/mL) |
| *Streptococcus salivarius* TOVE-R | 5% CO_2_ | blood agar supplemented with hemin (5.0 µg/mL), menadione (1.0 µg/mL) |
| *Streptococcus mutans* ATCC 20523 | 5% CO_2_ | blood agar supplemented with hemin (5.0 µg/mL), menadione (1.0 µg/mL) |

**Supplementary table 1:** Analysis of variance (ANOVA) followed by Tukey’s post hoc test for multiple comparisons of antibacterial assay.

| **Bacteria** | **Saliva** | **Time (sec)** | **Antiseptic** | **log_10_ CFU/mL** | **Mean difference compared to NaCl log_10_ CFU/mL** | **95% Cl difference compared to NaCl log_10_ CFU/mL** | **p-value compared to Control** | **p-value compared to CHX** |
| --- | --- | --- | --- | --- | --- | --- | --- | --- |
| *S. mitis* | No | 0 | Control | 8.24 ± 0.06 | / | / | / | / |
|  | No | 0 | CHX | 8.24 ± 0.06 | 0.00 | -0.08 – 0.08 | > 0.999 | / |
|  | No | 0 | EOS | 8.24 ± 0.06 | 0.00 | -0.08 – 0.08 | > 0.999 | > 0.999 |
|  | No | 10 | Control | 8.24 ± 0.06 | / | / | / | / |
|  | No | 10 | CHX | ≤ LOD | 7.24 | 7.16 – 7.32 | <0.001* | / |
|  | No | 10 | EOS | ≤ LOD | 7.24 | 7.16 – 7.32 | <0.001* | > 0.999 |
|  | No | 30 | Control | 8.24 ± 0.16 | / | / | / | / |
|  | No | 30 | CHX | ≤ LOD | 7.24 | 7.16 – 7.32 | <0.001* | / |
|  | No | 30 | EOS | ≤ LOD | 7.24 | 7.16 – 7.32 | <0.001* | > 0.999 |
|  | No | 60 | Control | 8.24 ± 0.16 | / | / | / | / |
|  | No | 60 | CHX | ≤ LOD | 7.24 | 7.16 – 7.32 | <0.001* | / |
|  | No | 60 | EOS | ≤ LOD | 7.24 | 7.16 – 7.32 | <0.001* | > 0.999 |
|  | Yes | 0 | Control | 9.18 ± 0.08 | / | / | / | / |
|  | Yes | 0 | CHX | 9.18 ± 0.08 | 0.00 | -0.22 – 0.22 | > 0.999 | / |
|  | Yes | 0 | EOS | 9.18 ± 0.08 | 0.00 | -0.22 – 0.22 | > 0.999 | > 0.999 |
|  | Yes | 10 | Control | 9.18 ± 0.08 | / | / | / | / |
|  | Yes | 10 | CHX | ≤ LOD | 8.18 | 7.96 – 8.40 | <0.001* | / |
|  | Yes | 10 | EOS | 4.94 ± 0.33 | 4.24 | 4.02 – 4.46 | <0.001* | <0.001^#^ |
|  | Yes | 30 | Control | 9.18 ± 0.08 | / | / | / | / |
|  | Yes | 30 | CHX | ≤ LOD | 8.18 | 7.96 – 8.40 | <0.001* | / |
|  | Yes | 30 | EOS | 3.86 ± 0.11 | 5.32 | 5.09 – 5.56 | <0.001* | <0.001^#^ |
|  | Yes | 60 | Control | 9.18 ± 0.08 | / | / | / | / |
|  | Yes | 60 | CHX | ≤ LOD | 8.18 | 7.96 – 8.40 | <0.001* | / |
|  | Yes | 60 | EOS | ≤ LOD | 8.18 | 7.96 – 8.40 | <0.001* | > 0.999 |
| *S. pyogenes* | No | 0 | Control | 6.01 ± 0.08 | / | / | / | / |
|  | No | 0 | CHX | 6.01 ± 0.08 | 0.00 | -0.58 – 0.58 | > 0.999 | / |
|  | No | 0 | EOS | 6.01 ± 0.08 | 0.00 | -0.58 – 0.58 | > 0.999 | > 0.999 |
|  | No | 10 | Control | 6.01 ± 0.08 | / | / | / | / |
|  | No | 10 | CHX | ≤ LOD | 5.01 | 4.66 – 5.38 | <0.001* | / |
|  | No | 10 | EOS | 2.18 ± 0.00 | 3.83 | 3.48 – 4.41 | <0.001* | <0.001^#^ |
|  | No | 30 | Control | 6.01 ± 0.08 | / | / | / | / |
|  | No | 30 | CHX | ≤ LOD | 5.01 | 4.66 – 5.38 | <0.001* | / |
|  | No | 30 | EOS | 2.54 ± 0.08 | 3.47 | 312 – 3.82 | <0.001* | <0.001^#^ |
|  | No | 60 | Control | 6.01 ± 0.08 | / | / | / | / |
|  | No | 60 | CHX | ≤ LOD | 5.01 | 4.66 – 5.38 | <0.001* | / |
|  | No | 60 | EOS | 1.39 ± 0.55 | 4.62 | 4.27 – 4.97 | <0.001* | 0.026^#^ |
|  | Yes | 0 | Control | 6.12 ± 0.07 | / | / | / | / |
|  | Yes | 0 | CHX | 6.12 ± 0.07 | 0.00 | -0.81 – 0.81 | > 0.999 | / |
|  | Yes | 0 | EOS | 6.12 ± 0.07 | 0.00 | -0.81 – 0.81 | > 0.999 | > 0.999 |
|  | Yes | 10 | Control | 6.12 ± 0.07 | / | / | / | / |
|  | Yes | 10 | CHX | ≤ LOD | 5.12 | 4.65 – 5.59 | <0.001* | / |
|  | Yes | 10 | EOS | 1.78 ± 0.55 | 4.34 | 3.87 – 4.08 | <0.001* | <0.001^#^ |
|  | Yes | 30 | Control | 6.12 ± 0.07 | / | / | / | / |
|  | Yes | 30 | CHX | ≤ LOD | 5.12 | 4.65 – 5.59 | <0.001* | / |
|  | Yes | 30 | EOS | ≤ LOD | 5.12 | 4.65 – 5.59 | <0.001* | > 0.999 |
|  | Yes | 60 | Control | 6.12 ± 0.07 | / | / | / | / |
|  | Yes | 60 | CHX | ≤ LOD | 5.12 | 4.65 – 5.59 | <0.001* | / |
|  | Yes | 60 | EOS | 1.39 ± 0.55 | 4.73 | 4.26 – 5.20 | <0.001* | 0.116 |
| *H. influenzae* | No | 0 | Control | 6.10 ± 0.23 | / | / | / | / |
|  | No | 0 | CHX | 6.10 ± 0.23 | 0.00 | -0.31 – 0.31 | > 0.999 | / |
|  | No | 0 | EOS | 6.10 ± 0.23 | 0.00 | -0.31 – 0.31 | > 0.999 | > 0.999 |
|  | No | 10 | Control | 6.10 ± 0.23 | / | / | / | / |
|  | No | 10 | CHX | ≤ LOD | 5.10 | 4.77 –5.431 | <0.001* | / |
|  | No | 10 | EOS | ≤ LOD | 5.10 | 4.77 –5.431 | <0.001* | > 0.999 |
|  | No | 30 | Control | 6.10 ± 0.23 | / | / | / | / |
|  | No | 30 | CHX | ≤ LOD | 5.10 | 4.77 –5.431 | <0.001* | / |
|  | No | 30 | EOS | ≤ LOD | 5.10 | 4.77 –5.431 | <0.001* | > 0.999 |
|  | No | 60 | Control | 6.10 ± 0.23 | / | / | / | / |
|  | No | 60 | CHX | ≤ LOD | 5.10 | 4.77 –5.431 | <0.001* | / |
|  | No | 60 | EOS | ≤ LOD | 5.10 | 4.77 –5.431 | <0.001* | > 0.999 |
|  | Yes | 0 | Control | 4.97 ± 0.08 | / | / | / | / |
|  | Yes | 0 | CHX | 4.97 ± 0.08 | 0.00 | -0.81 – 0.81 | > 0.999 | / |
|  | Yes | 0 | EOS | 4.97 ± 0.08 | 0.00 | -0.81 – 0.81 | > 0.999 | > 0.999 |
|  | Yes | 10 | Control | 4.97 ± 0.08 | / | / | / | / |
|  | Yes | 10 | CHX | ≤ LOD | 3.97 | 3.40 – 4.54 | <0.001* | / |
|  | Yes | 10 | EOS | 1.88 ± 0.64 | 3.09 | 2.52 – 3.66 | <0.001* | 0.002^#^ |
|  | Yes | 30 | Control | 4.97 ± 0.08 | / | / | / | / |
|  | Yes | 30 | CHX | ≤ LOD | 3.97 | 3.40 – 4.54 | <0.001* | / |
|  | Yes | 30 | EOS | ≤ LOD | 3.97 | 3.40 – 4.54 | <0.001* | > 0.999 |
|  | Yes | 60 | Control | 4.97 ± 0.08 | / | / | / | / |
|  | Yes | 60 | CHX | ≤ LOD | 3.97 | 3.40 – 4.54 | <0.001* | / |
|  | Yes | 60 | EOS | 1.49 ± 0.70 | 3.48 | 2.91 – 4.05 | <0.001* | 0.102 |
| *S. pneumoniae* | No | 0 | Control | 7.60 ± 0.18 | / | / | / | / |
|  | No | 0 | CHX | 7.60 ± 0.18 | 0.00 | -0.25 – 0.25 | > 0.999 | / |
|  | No | 0 | EOS | 7.60 ± 0.18 | 0.00 | -0.25 – 0.25 | > 0.999 | > 0.999 |
|  | No | 10 | Control | 7.60 ± 0.18 | / | / | / | / |
|  | No | 10 | CHX | ≤ LOD | 6.60 | 6.38 – 6.86 | <0.001* | / |
|  | No | 10 | EOS | 2.87 ± 0.07 | 4.73 | 4.48– 4.98 | <0.001* | <0.001^#^ |
|  | No | 30 | Control | 7.60 ± 0.18 | / | / | / | / |
|  | No | 30 | CHX | ≤ LOD | 6.60 | 6.38 – 6.86 | <0.001* | / |
|  | No | 30 | EOS | ≤ LOD | 6.60 | 6.38 – 6.86 | <0.001* | > 0.999 |
|  | No | 60 | Control | 7.60 ± 0.18 | / | / | / | / |
|  | No | 60 | CHX | ≤ LOD | 6.60 | 6.38 – 6.86 | <0.001* | / |
|  | No | 60 | EOS | ≤ LOD | 6.60 | 6.38 – 6.86 | <0.001* | > 0.999 |
|  | Yes | 0 | Control | 7.52 ± 0.02 | / | / | / | / |
|  | Yes | 0 | CHX | 7.52 ± 0.02 | 0.00 | -1.02 – 1.02 | > 0.999 | / |
|  | Yes | 0 | EOS | 7.52 ± 0.02 | 0.00 | -1.02 – 1.02 | > 0.999 | > 0.999 |
|  | Yes | 10 | Control | 7.52 ± 0.02 | / | / | / | / |
|  | Yes | 10 | CHX | ≤ LOD | 6.52 | 5.81 – 7.22 | <0.001* | / |
|  | Yes | 10 | EOS | 1.78 ± 0.55 | 5.74 | 5.04 – 6.44 | <0.001* | 0.028^#^ |
|  | Yes | 30 | Control | 7.52 ± 0.02 | / | / | / | / |
|  | Yes | 30 | CHX | ≤ LOD | 6.52 | 5.81 – 7.22 | <0.001* | / |
|  | Yes | 30 | EOS | ≤ LOD | 6.52 | 5.81 – 7.22 | <0.001* | > 0.999 |
|  | Yes | 60 | Control | 7.52 ± 0.02 | / | / | / | / |
|  | Yes | 60 | CHX | ≤ LOD | 6.52 | 5.81 – 7.22 | <0.001* | / |
|  | Yes | 60 | EOS | 1.75 ± 1.06 | 5.77 | 5.07 – 6.47 | <0.001* | 0.035^#^ |

The reference category is Control. Control – saline, EOS−electrolyzed saline, CHX – chlorhexidine digluconate, LOD – limit of detection (≤ 10 CFU/mL); * – statistical significance compared to Control at α = 0.05; #– statistical significance compared to CHX at α = 0.05.

**Supplementary table 2:** Generalized linear mixed model of bacterial dental spray deposition.

|  | **OR** | **95% CI** |
| --- | --- | --- |
| Intercept | 2294.000 | [21, 253947] |
| CHX | 0.000 | [0,0] |
| EOS | 0.000 | [0, 0.007] |
| distance | 1.844 | [0.107, 31.813] |
| CHX: distance | 0.771 | [0.045, 13.299] |
| EOS: distance | 0.406 | [0.023, 7.032] |
| distance slope CHX | 1.421 | [1.380, 1.463] |
| distance slope EOS | 0.749 | [0.661, 0.850] |
| sigma | 0.087 | [0.011, 0.673] |

Note: The reference category is saline. EOS−electrolyzed saline, CHX – chlorhexidine digluconate

**Supplementary table 3:** Multiple linear regression of bacterial dental spray deposition CFU/cm^2^ quantification.

Model: CFU/cm^2^ = Intercept + mouthrinse + distance + position

| **Variable** | **Estimate** | **95% CI** | **p value** |
| --- | --- | --- | --- |
| Intercept | 93.55 | [85.52, 101.6] | <0.0001* |
| CHX | -76.15 | [-83.37, -68.93] | <0.0001* |
| EOS | -85.71 | [-92.93, -78.49] | <0.0001* |
| distance | -0.71 | [-1.23, -0.19] | 0.0076* |
| position right | -2.40 | [-9.66, 4.86] | 0.5153 |
| position left | 0.96 | [-6.31, 8.22] | 0.7952 |

Note: The reference category is saline and central position. EOS−electrolyzed saline, CHX – chlorhexidine digluconate; * – statistical significance at α = 0.05.

**Supplementary table 4:** Multiple linear regression of bacterial dental spray deposition CFU/cm^2^ quantification.

Model: CFU/cm^2^ = Intercept + mouthrinse + distance + position + mouthrinse : distance

| **Variable** | **Estimate** | **95% CI** | **p value** |
| --- | --- | --- | --- |
| Intercept | 121.8 | [112.6, 130.9] | <0.0001* |
| CHX | -124.7 | [-137.1, -112.3] | <0.0001* |
| EOS | -121.8 | [-134.1, -109,4] | <0.0001* |
| distance | -3.40 | [-4.13, -2.66] | <0.0001* |
| position right | -2.40 | [-8.38, 3.58] | 0.4298 |
| position left | 0.96 | [-5.03, 6.94] | 0.7527 |
| CHX : distance | 4,62 | [3.59, 5.66] | <0.0001* |
| EOS : distance | 3.43 | [2.62, 5.66] | <0.0001* |

Note: The reference category is saline and central position. EOS−electrolyzed saline, CHX – chlorhexidine digluconate; * – statistical significance at α = 0.05.

**Supplementary table 5:** Analysis of variance (ANOVA) followed by Dunnett’s post hoc test for multiple comparisons of biocompatibility assay.

| **Cells** | **Antiseptic** | **Dilution** | **Mean ± SD (%)** | **Mean difference compared to PBS** | **95% Cl difference compared to PBS** | **p-value** |
| --- | --- | --- | --- | --- | --- | --- |
| hGF | PBS | no dilution | 104.2 ± 8.7 |  |  |  |
|  | PBS | 1:2 | 86.4 ± 8.7 | / | / | / |
|  | PBS | 1:4 | 91.4 ± 8.7 | / | / | / |
|  | PBS | 1:8 | 87.0 ± 8.7 | / | / | / |
|  | PBS | 1:16 | 89.8 ± 8.7 | / | / | / |
|  | PBS | 1:32 | 74.8 ± 8.7 | / | / | / |
|  | CHX | no dilution | 1.4 ± 0.1 | 102.9 | 83.9 – 121.9 | < 0.001* |
|  | CHX | 1:2 | 1.9 ± 0.2 | 84.6 | 65.6 – 103.6 | < 0.001* |
|  | CHX | 1:4 | 2.1 ± 0.1 | 89.3 | 70.3 – 108.3 | < 0.001* |
|  | CHX | 1:8 | 2.5 ± 0.5 | 84.5 | 65.5 – 103.5 | < 0.001* |
|  | CHX | 1:16 | 32.1 ± 6.6 | 57.6 | 38.6 – 76.6 | < 0.001* |
|  | CHX | 1:32 | 70.8 ± 7.0 | 4.0 | -15.0 – 23.0 | 0.994 |
|  | EOS | no dilution | 88.5 ± 8.0 | 15.71 | -3.3 – 34.7 | 0.150 |
|  | EOS | 1:2 | 113.3 ± 11.5 | -26.9 | -45.9 – 7.9 | 0.002* |
|  | EOS | 1:4 | 93.5 ± 7.9 | -2.1 | -21.1 – 16.9 | > 0.999 |
|  | EOS | 1:8 | 110.6 ± 10.5 | -23.6 | -42.6 – 4.6 | 0.008* |
|  | EOS | 1:16 | 97.3 ± 14.6 | -7.5 | -26.5 – 11.5 | 0.846 |
|  | EOS | 1:32 | 72.3 ± 6.0 | 2.5 | -16.5 – 21.5 | > 0.999 |
| hOK | PBS | no dilution | 90.1 ± 5.2 | / | / | / |
|  | PBS | 1:2 | 97.6 ± 5.2 | / | / | / |
|  | PBS | 1:4 | 101.4 ± 5.2 | / | / | / |
|  | PBS | 1:8 | 89.3 ± 5.2 | / | / | / |
|  | PBS | 1:16 | 92.7 ± 5.2 | / | / | / |
|  | PBS | 1:32 | 86.2 ± 5.2 | / | / | / |
|  | CHX | no dilution | 0.6 ± 0.2 | 89.5 | 72.1 – 106.9 | < 0.001* |
|  | CHX | 1:2 | 0.2 ± 0.4 | 97.4 | 80.0 – 114.8 | < 0.001* |
|  | CHX | 1:4 | 0.3 ± 0.13 | 101.1 | 83.7 – 118.4 | < 0.001* |
|  | CHX | 1:8 | 2.2 ± 1.0 | 87.0 | 69.7 – 104.4 | < 0.001* |
|  | CHX | 1:16 | 31.8 ± 9.3 | 60.9 | 43.5 – 78.3 | < 0.001* |
|  | CHX | 1:32 | 60.0 ± 18.5 | 26.2 | 8.6 – 43.6 | < 0.001* |
|  | EOS | no dilution | 88.5 ± 8.0 | 35.13 | 17.8 – 52.5 | < 0.001* |
|  | EOS | 1:2 | 113.3 ± 11.5 | 19.7 | 2.3 – 37.0 | 0.019* |
|  | EOS | 1:4 | 93.5 ± 7.9 | 19.3 | -1.9 – 36.7 | 0.022* |
|  | EOS | 1:8 | 110.6 ± 10.5 | 0.8 | -16.6 – 18.1 | > 0.999 |
|  | EOS | 1:16 | 97.3 ± 14.6 | 15.3 | -2.1 – 32.6 | 0.109 |
|  | EOS | 1:32 | 72.3 ± 6.0 | 11.6 | -5.8 – 29.0 | 0.342 |

Note: The reference category is phosphate buffered saline. hGF− human gingival fibroblasts, hOK – human oral keratinocytes, EOS−electrolyzed saline, CHX – chlorhexidine digluconate, phosphate buffered saline (PBS); * – statistical significance at α = 0.05.

**Supplementary figure 1:**

Spatial representation of dental spray bacterial deposition assay during a simulated dental procedure using ultrasonic scaler. Panels A-C show SD for quantification of bacterial load (CFUs/cm^2^) for saline (Control), chlorhexidine digluconate (CHX), and electrolyzed saline (EOS) represented on a red gradient.
